# Experimentally‐induced anti‐myeloperoxidase vasculitis does not require properdin, MASP‐2 or bone marrow‐derived C5

**DOI:** 10.1002/path.4754

**Published:** 2016-08-22

**Authors:** Simon J Freeley, Reena J Popat, Kiran Parmar, Martin Kolev, Beverley J Hunt, Cordula M Stover, Willhelm Schwaeble, Claudia Kemper, Michael G Robson

**Affiliations:** ^1^ Division of Transplantation Immunology and Mucosal Biology, Faculty of Life Sciences and Medicine King's College London London UK; ^2^ Thrombosis and Vascular Biology Guy's and St Thomas' NHS Foundation Trust London UK; ^3^ Department of Infection, Immunity and Inflammation University of Leicester Leicester UK

**Keywords:** complement, vasculitis, inflammation autoimmune

## Abstract

Anti‐neutrophil cytoplasmic antibody vasculitis is a systemic autoimmune disease with glomerulonephritis and pulmonary haemorrhage as major clinical manifestations. The name reflects the presence of autoantibodies to myeloperoxidase and proteinase‐3, which bind to both neutrophils and monocytes. Evidence of the pathogenicity of these autoantibodies is provided by the observation that injection of anti‐myeloperoxidase antibodies into mice causes a pauci‐immune focal segmental necrotizing glomerulonephritis which is histologically similar to the changes seen on renal biopsy in patients. Previous studies in this model have implicated the alternative pathway of complement activation and the anaphylatoxin C5a. Despite this progress, the factors that initiate complement activation have not been defined. In addition, the relative importance of bone marrow‐derived and circulating C5 is not known. This is of interest given the recently identified roles for complement within leukocytes. We induced anti‐myeloperoxidase vasculitis in mice and confirmed a role for complement activation by demonstrating protection in C3‐deficient mice. We showed that neither MASP‐2‐ nor properdin‐deficient mice were protected, suggesting that alternative pathway activation does not require properdin or the lectin pathway. We induced disease in bone marrow chimaeric mice and found that circulating and not bone marrow‐derived C5 was required for disease. We have therefore excluded properdin and the lectin pathway as initiators of complement activation and this means that future work should be directed at other potential factors within diseased tissue. In addition, in view of our finding that circulating and not bone marrow‐derived C5 mediates disease, therapies that decrease hepatic C5 secretion may be considered as an alternative to those that target C5 and C5a. © 2016 The Authors. *The Journal of Pathology* published by John Wiley & Sons Ltd on behalf of Pathological Society of Great Britain and Ireland.

## Introduction

Anti‐neutrophil cytoplasmic antibody (ANCA) vasculitis is a systemic disease causing crescentic glomerulonephritis and pulmonary haemorrhage. Other clinical features include disease affecting skin, the nervous system, and upper airways 1. The name ANCA vasculitis reflects the fact that it is characterized by autoantibodies against neutrophils 2, 3, although they also bind to monocytes. The antigenic targets of the autoantibodies have been identified as myeloperoxidase (MPO) or proteinase 3 (PR3) 4, 5 and these enzymes are indeed found within granules in both neutrophils and monocytes. In addition to their clinical utility, the suggestion that these autoantibodies were pathogenic came from *in vitro* studies in which IgG from patients with anti‐MPO or anti‐PR3 antibodies activates neutrophils to undergo respiratory burst and degranulation 6. Many subsequent studies have supported this observation (reviewed in ref 7). However, it was not until 2002 that *in vivo* evidence of pathogenicity was obtained. Anti‐MPO antibodies raised in MPO‐deficient mice were shown to cause a focal necrotizing crescentic glomerulonephritis when injected into wild‐type mice 8.

In this murine anti‐MPO model, both the histological and the clinical features of glomerulonephritis closely mirror the situation in patients. The crescentic glomerulonephritis is focal and segmental, affecting segments of some glomeruli and not others, and also shows necrosis with a lack of immune deposits. All of these features recapitulate characteristics of the histology seen in clinical renal biopsy samples. Furthermore, proteinuria is relatively mild and not in the nephrotic range, as is the case for patients. In addition to providing evidence of pathogenicity, the murine anti‐MPO model has become established as a preclinical model, which is useful for understanding mechanisms and developing therapies in ANCA vasculitis. Previous work using this model has suggested that the alternative pathway (AP) is important, as mice deficient in factor B, but not C4, were protected 9. C5‐deficient mice are also protected, and treatment with an anti‐C5 monoclonal antibody inhibited disease 10, providing evidence of a role for C5. Furthermore, MPO‐deficient mice immunized with MPO and transplanted with bone marrow from C5a receptor‐deficient mice were protected from disease, compared with mice that received wild‐type bone marrow, suggesting that the anaphylatoxin C5a is the key mediator 11. This work has recently been extended with evidence of therapeutic efficacy for the C5a receptor antagonist CCX168 in mice in which the C5a receptor has been replaced with the human equivalent 12. In addition, disease was exacerbated in mice defective in the second C5a receptor C5L2 12. This work in the anti‐MPO model has led to clinical trials in which CCX168 is currently being tested in patients with ANCA vasculitis (trial registrations NCT01363388 and NCT02222155).

The trigger leading to AP activation has not been identified and this raises an important question given the interest in therapies targeting the complement pathway in ANCA vasculitis. Properdin is established as a key stabilizer of the AP but recent data have also shown that it may bind to apoptotic cells and initiate AP activation 13, 14. Another candidate for initiating AP activation is the lectin pathway. Recent work has shown that C4 is not required for lectin pathway activation 15, and so we considered that the lectin pathway may be important in initiating complement activation, with subsequent amplification by the AP. Therefore, in order to explore the initial events leading to complement activation, we studied both properdin‐deficient and MBL‐associated serine protease 2 (MASP‐2)‐deficient mice in the anti‐MPO model. Finally, in view of the recently discovered importance of intracellular complement components 16, 17, 18, 19, 20, 21, we examined the relative importance of bone marrow‐derived and circulating C5 in anti‐MPO vasculitis.

## Materials and methods

### Mice

Wild‐type C57BL/6 mice were from Harlan (Bicester, Oxon, UK). MASP‐2‐ 15, C3‐ 22, properdin‐ 23, and C5‐deficient mice 24 have been previously described. We confirmed that mice were C5‐deficient by testing serum using a mouse C5 ELISA duoset (R&D Systems, Abingdon, Oxfordshire, UK). MASP‐2‐deficient mice were provided by Omeros Corp (Seattle, WA, USA). Properdin‐deficient mice were backcrossed more than 16 generations, and all others were backcrossed at least ten generations to C57BL/6. MASP‐2 × C3‐deficient mice were generated, with homozygosity for the disrupted MASP‐2 allele confirmed by PCR and the absence of serum C3 confirmed by ELISA 25. Bone marrow chimaeras were made as described previously 26. A parallel cohort of chimaeras was made between wild‐type C57BL/6 mice and C57BL/6 mice congenic for CD45.1 (B6.SJL‐*Ptprc^a^ Pepc^b^*/BoyJ). Chimaerism was determined by flow cytometry for CD45.2 on whole blood with clone 104 from eBioscience (Hatfield, UK), and chimaerism was more than 90% in all cases (*n* = 7). Animal experiments complied with the Animals (Scientific Procedures) Act 1986 and Home Office and local regulations.

### Induction of anti‐MPO crescentic glomerulonephritis

Anti‐MPO antibody was generated and glomerulonephritis induced as previously described 27, with minor modifications. Day 0 denotes the day that 2 mg of anti‐MPO IgG was injected. In all experiments apart from those with C5 bone marrow chimaeras, 30 µg of pegylated GCSF (Neulasta, Amgen, Cambridge) was given subcutaneously on days −8, −4, 0 and 4, and 10 µg of LPS (from *E. coli* serotype 0111 B4; Enzo Life Sciences, Exeter, UK) was given intraperitoneally on days 0 and 3. In the C5 bone marrow chimaera experiment, anti‐MPO IgG was given intraperitoneally and 30 µg of pegylated GCSF was given subcutaneously on days −4 and 0. 2.5 µg of LPS was given intraperitoneally on day 0 only. Blood was taken from the saphenous vein on day −1 to measure circulating neutrophils by flow cytometry 27 or baseline serum creatinine by mass spectrometry 28. For the experiments with MASP‐2‐ and properdin‐deficient mice, metabolic cages were used to collect urine in the last 24 h of the experiment. In other experiments, spot urine was taken on day −1 and on day 6 for the urine albumin creatinine ratio. In all experiments, mice were killed on day 7.

### Assessment of disease

Urine creatinine was measured using a commercial creatinase assay (Diazyme, Dresden, Germany) based on the manufacturer's instructions, with a standard curve generated for all assays. Histology, serum creatinine and urine albumin analysis were as described previously 27, 28. Crescent formation was defined as at least two layers of non‐epithelial cells in Bowman's space. Immunofluorescence staining for MBL and C3 was performed using tissue fixed with phosphate–lysine–periodate prior to freezing and sectioning. For MBL, clone 16A8 (Hycult Biotechnology, Uden, The Netherlands) was used and for C3, clone RMC11H9 (Cedarlane, Burlington, Ontario, Canada). Detection was with DyLight 488 mouse anti‐rat IgG (Jackson ImmunoResearch Laboratories, West Grove, PA, USA). Sections stained with secondary antibody only were included as controls and were negative (supplementary material, Figure S1). Fibrin staining was produced using a FITC‐labelled rabbit anti‐human fibrinogen/fibrin antibody (Dako, Cambridge, UK) which cross‐reacted with mouse fibrinogen/fibrin. For C3, glomerular fluorescence intensity was quantified using ImageJ software (NIH, USA). For MBL and fibrin quantification, the software used was Cell (Olympus, Southend‐on‐Sea, UK). A minimum of 20 glomeruli per sample were included in all cases.

### Coagulation assays

Blood (900 µl) was taken by intracardiac puncture under terminal anaesthesia into a 1 ml syringe containing 100 µl of 3.2% trisodium citrate (Sigma, Poole, UK). Plasma was double spun (800 *g*), aliquoted, and frozen until analysed. Thrombin generation was monitored with a Fluoroskan Ascent Thrombinoscope (Thermo Electron Corporation, Paisley, UK) and Thrombinoscope software version 5. Samples were run at a dilution of 1 in 3 and at 33 °C 29. Clauss fibrinogen was measured using the ACL300R analyser, and using the HemosIL Fibrinogen‐C reagent by Werfen UK (Warrington, Cheshire, UK). Prothrombin fragment 1.2 was measured by ELISA using an Enzygost F1 + 2 Micro kit from Sysmex UK Ltd (Wymbush, Milton Keynes, UK).

### Neutrophil activation

Human neutrophils were isolated from healthy donors (ethical approval NRES committee London – London Bridge 09/H084/72) as previously described 27 and primed with 2 ng/ml TNFα for 15 min at 37 °C and stimulated with antibody or fMLP for 60 min. Monoclonal mouse IgG1 anti‐MPO was clone 266.6 K2 (IQ Products Company, Houston, TX, USA), anti‐PR3 was clone WGM2 (Hycult Biotech, Uden, Netherlands), and control mouse IgG1 was anti‐TNP clone 107.3 (BD Biosciences, Oxford, UK). Monoclonals were used at 5 µg/ml and fMLP at 100 ng/ml. The reaction was stopped with a 30‐fold volume of cold HBSS with HEPES (Sigma). The C5 antibody was a rabbit polyclonal and C5a was clone 2952 (PE conjugate), both from Abcam (Cambridge, UK). The secondary antibody for C5 staining was goat anti‐rabbit IgG (Alexa Fluor 488 conjugate) from Invitrogen (Carlsbad, CA, USA). Cells were permeabilized with cytofix/cytoperm (BD Biosciences). A FACS Canto flow cytometer (BD Biosciences) and FACsDIVA software (BD Biosciences) was used. C5a was assayed using cytometric beads (BD Biosciences).

### Statistics

GraphPad Prism version 5 (GraphPad Software Inc, La Jolla, CA, USA), with Student's *t*‐test, was used where two groups were compared. If more than two groups were compared, a one‐way ANOVA with Tukey's post‐test was used. Some data were logarithmically transformed before analysis if the variances of the groups were significantly different.

## Results

### Properdin‐deficient mice are not protected in anti‐MPO vasculitis

**Figure 1:**
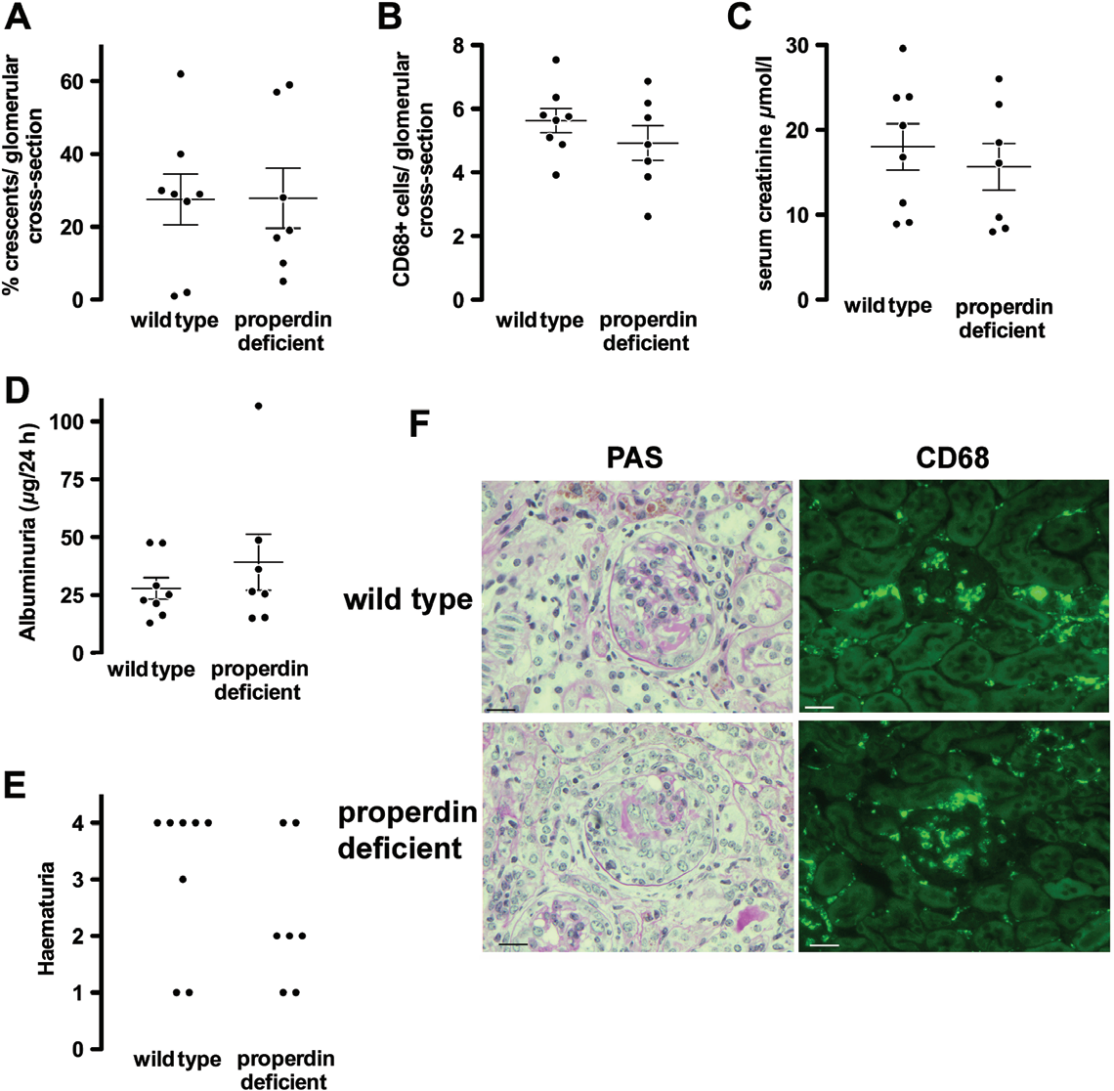
Histological and functional readouts of anti‐MPO vasculitis in properdin‐deficient mice compared with wild types. (A, B) Histological readouts of glomerular crescents and glomerular CD68+ macrophages. (C–E) Functional readouts of serum creatinine, albuminuria, and haematuria. Each symbol represents a separate mouse. (F) Representative renal histology showing periodic acid Schiff (PAS)‐stained sections and immunofluorescence staining for CD68+ macrophages from mice with anti‐MPO vasculitis. Scale bars = 20 µm. Error bars are mean ± SEM.

Because previous work had shown that the AP was important in anti‐MPO vasculitis, we wondered if properdin was essential and perhaps could initiate complement activation. We therefore studied properdin‐deficient mice with the aim of confirming and extending our understanding of AP activation in anti‐MPO vasculitis. We found that both histological and functional parameters of disease severity were similar to wild types as shown in Figure 1A–E. Figure 1F shows representative histology and CD68+ macrophage staining. Circulating neutrophil counts were the same in both groups after GCSF treatment (supplementary material, Table S1). These data showed that properdin is not essential for AP activation in anti‐MPO vasculitis, and that the trigger for AP activation involves other mechanisms.

### MASP‐2‐deficient mice develop increased disease in the anti‐MPO vasculitis model

**Figure 2:**
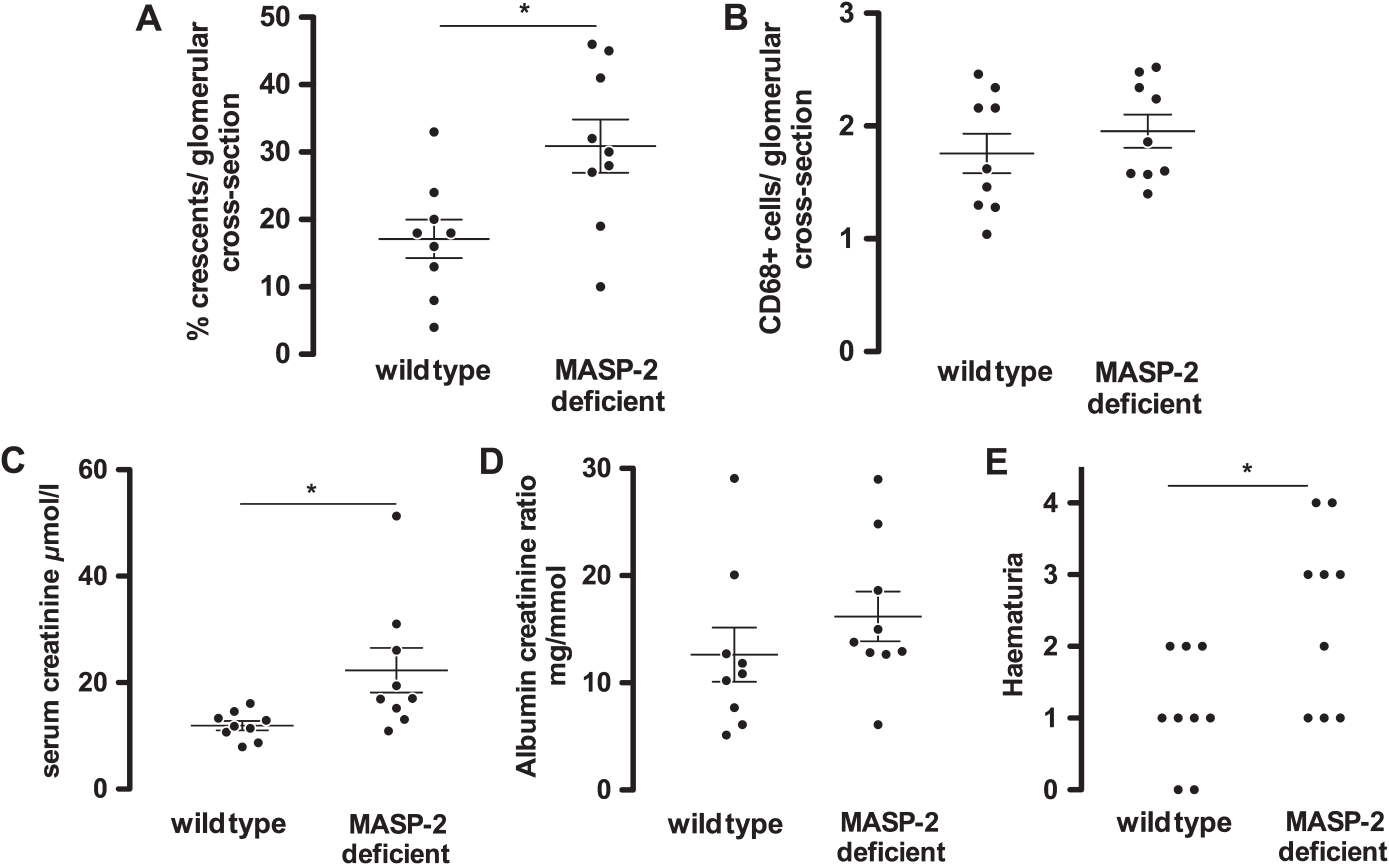
Histological and functional readouts of anti‐MPO vasculitis in MASP‐2‐deficient mice compared with wild types. (A, B) Histological readouts of glomerular crescents and glomerular CD68+ macrophages. (C–E) Functional readouts of serum creatinine, albuminuria, and haematuria. At baseline, there was no haematuria detected in any of the wild‐type or MASP‐2‐deficient mice. Each symbol represents a separate mouse. *p < 0.05. Error bars are mean ± SEM.

Since properdin was not required, we considered that lectin pathway activation may initiate complement activation independently of C4 15. This might then be followed by amplification via the AP. We first induced crescentic glomerulonephritis in mice by injecting anti‐MPO antibody and compared disease severity at day 7 in MASP‐2‐deficient mice with wild types. MASP‐2‐deficient mice had significantly more disease, both functionally and histologically, than wild types, as shown in Figure 2. MASP‐2‐deficient mice had more glomerular crescents, more haematuria, and a higher serum creatinine. We did not find a difference in albuminuria, and the trend towards increased glomerular CD68+ macrophages did not reach significance. These findings were not due to differences in the clearance of anti‐MPO antibody as levels were the same at the end of the experiment (supplementary material, Figure S2). They were also not due to differences in circulating neutrophil counts following GCSF treatment (supplementary material, Table S1). Overall, these data demonstrated that MASP‐2‐deficient mice are not protected from crescentic glomerulonephritis induced by anti‐MPO antibodies and are in fact predisposed to more severe disease. Both properdin and the lectin pathway are therefore excluded as the initiator of AP activation in anti‐MPO vasculitis.

### MASP‐2/C3 double‐deficient‐ and C3‐deficient mice are protected from disease

**Figure 3:**
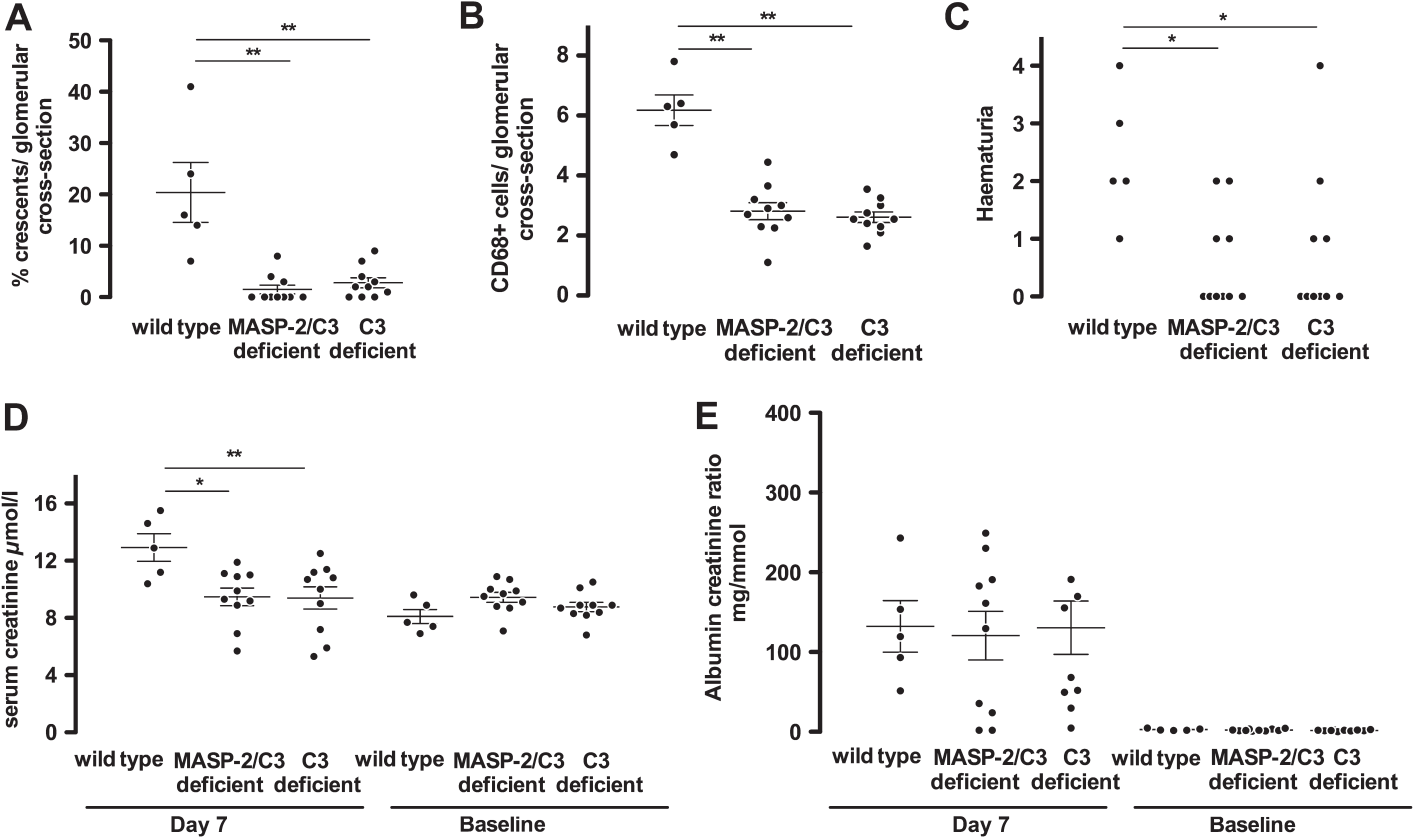
Histological and functional readouts of anti‐MPO vasculitis in mice deficient in both MASP‐2 and C3, compared with mice deficient in C3 and wild‐type mice. (A, B) Histological readouts of glomerular crescents and glomerular CD68+ macrophages. (C–E) Functional readouts of serum creatinine, albuminuria, and haematuria. Each symbol represents a separate mouse. *p < 0.05; **p < 0.01. Error bars are mean ± SEM.

**Figure 4:**
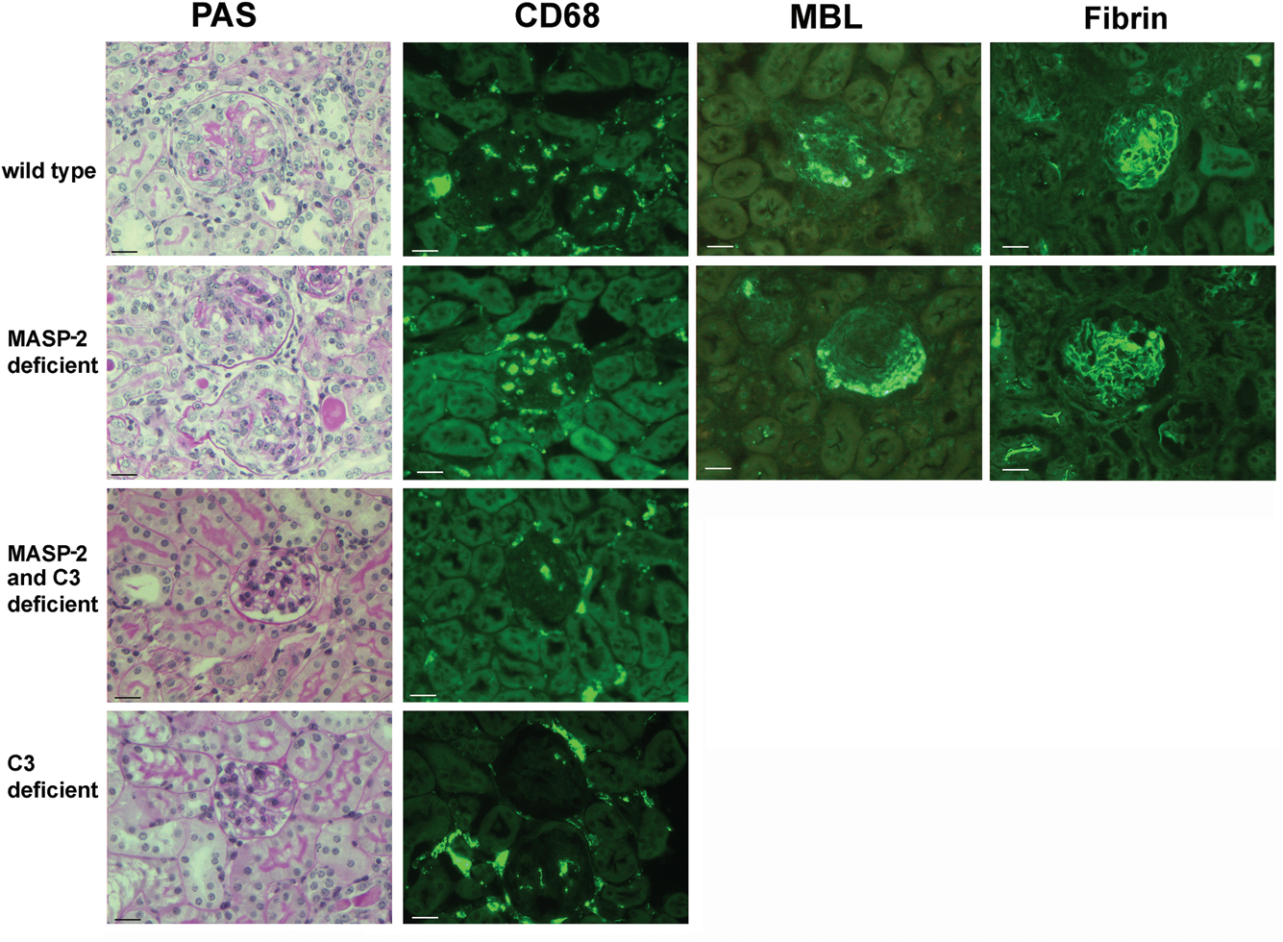
(A) Representative renal histology showing periodic acid Schiff (PAS)‐stained sections and immunofluorescence staining for CD68+ macrophages from mice with anti‐MPO vasculitis. The strains shown include wild types and MASP‐2‐, MASP‐2/C3‐, and C3‐deficient mice. For wild‐type and MASP‐2‐deficient mice, MBL and fibrin deposition is also shown and there were no differences between the two groups. Data for quantification of MBL and fibrin deposition are shown in supplementary material, Figure S5. Scale bars = 20 µm.

The reason for the increased disease severity in MASP‐2‐deficient mice was not clear; it was not due to a difference in anti‐MPO antibody remaining in the circulation at day 7 (supplementary material, Figure S2). We therefore sought to examine if the increased disease depended on complement activation and compared disease in wild‐type mice and mice deficient in both C3 and MASP‐2 with mice deficient in C3. As shown in Figure 3, there was very mild disease in both C3‐deficient and MASP‐2/C3 double‐deficient mice. There was no difference between these groups in any of the histological or functional parameters examined, and both had significantly less disease than wild‐type mice. This meant that we were unable to determine if the increase in disease, in MASP‐2‐deficient mice compared with wild types, depended on C3 activation with this experiment. However, circulating C3 levels in untreated MASP‐2‐deficient mice did not differ from wild types (supplementary material, Figure S3). In addition, glomerular deposition of C3 in MASP‐2‐deficient mice with anti‐MPO vasculitis was similar to that in wild‐type mice (supplementary material, Figure S3). This suggested that there was no major dysregulation of the AP of complement in MASP‐2‐deficient mice. Figure 4 shows representative histology and immunofluorescence staining for CD68+ macrophages in wild‐type, MASP‐2‐deficient, MASP‐2/C3‐deficient, and C3‐deficient mice. Also shown in Figure 4 is staining for MBL, which demonstrates deposition of MBL, and by inference MASP‐2, within diseased glomeruli. There was no difference in MBL deposition between the groups (supplementary material, Figure S4). Circulating neutrophil counts were the same in all groups after GCSF treatment (supplementary material, Table S1). These data show that both MASP‐2/C3‐deficient and C3‐deficient mice are protected from disease. The phenotype of C3‐deficient mice has not previously been reported in the anti‐MPO model, and these data confirm a central role for complement in this model in our hands.

### Evidence for increased prothrombin activation in MASP‐2‐deficient mice

**Figure 5:**
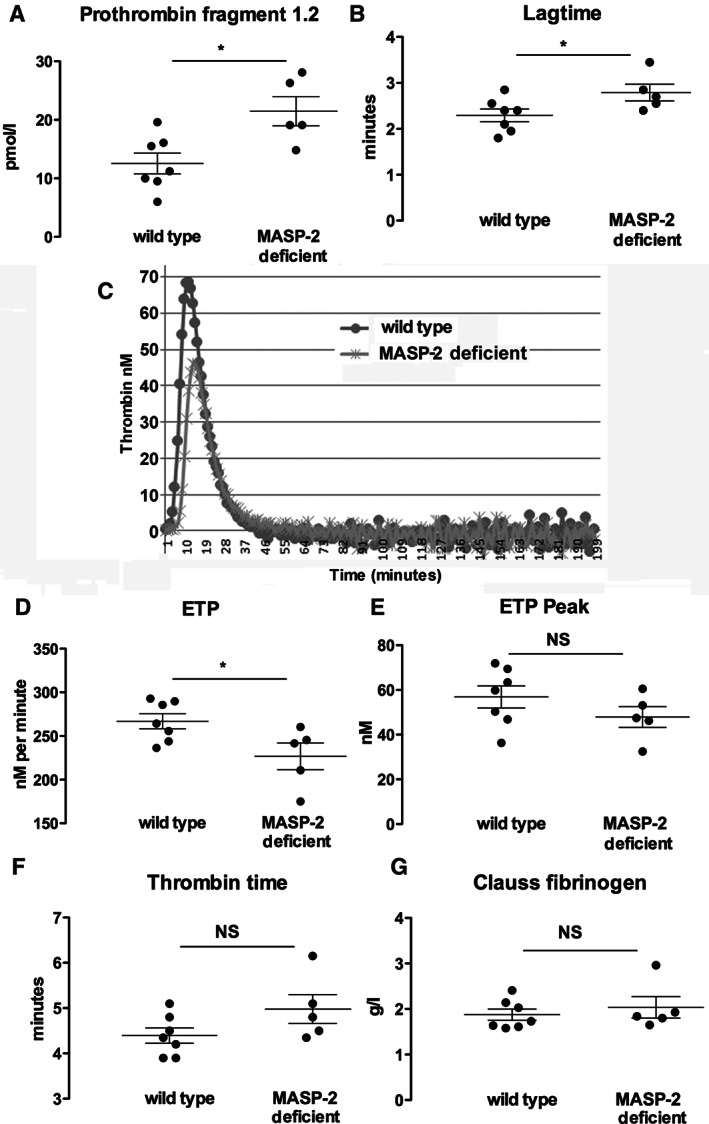
Coagulation parameters in plasma from untreated wild‐type or MASP‐2‐deficient mice. Each graph represents the parameter indicated, with panel C showing typical profiles for thrombin generation over time. Each symbol represents plasma from a separate mouse. *p < 0.05. Error bars are mean ± SEM.

Previous data have shown cross‐talk between MASP‐2 and the coagulation pathway 30. Since fibrin generation is an essential feature of glomerular crescent formation, we wondered if the increased disease might be explained by activation of coagulation predisposing to fibrin deposition in MASP‐2‐deficient mice. We confirmed this by measuring prothrombin fragment 1.2, which is a breakdown product of prothrombin activation and which was increased in the plasma of untreated MASP‐2 mice compared with wild type. This provided evidence of increased prothrombin activation (Figure 5A). We then measured residual thrombin generation in the plasma of MASP‐2‐deficient mice compared with wild type. Figure 5B shows a typical profile of thrombin generation over time from both a wild‐type and a MASP‐2‐deficient mouse. The data shown in Figure 4C–E show that although there was no difference in the peak thrombin generation, MASP‐2‐deficient mice had an increase in the lag time and a decrease in the overall endogenous thrombin potential (ETP). No significant difference in thrombin time or fibrinogen concentration was seen, but these are crude tests compared with the sensitivity of prothrombin fragment 1.2 and thrombin generation (Figure 5F, G). The decrease in ETP suggested a reduction in residual thrombin generation in plasma, due to consumption of prothrombin, and a situation analogous to mild disseminated intravascular coagulation. Despite these findings, we were unable to detect a difference in fibrin deposition in glomeruli in MASP‐2‐deficient compared with wild‐type mice (Figure 4 and supplementary material, Figure S4).

### Neutrophils contain intracellular C5 and C5a

**Figure 6:**
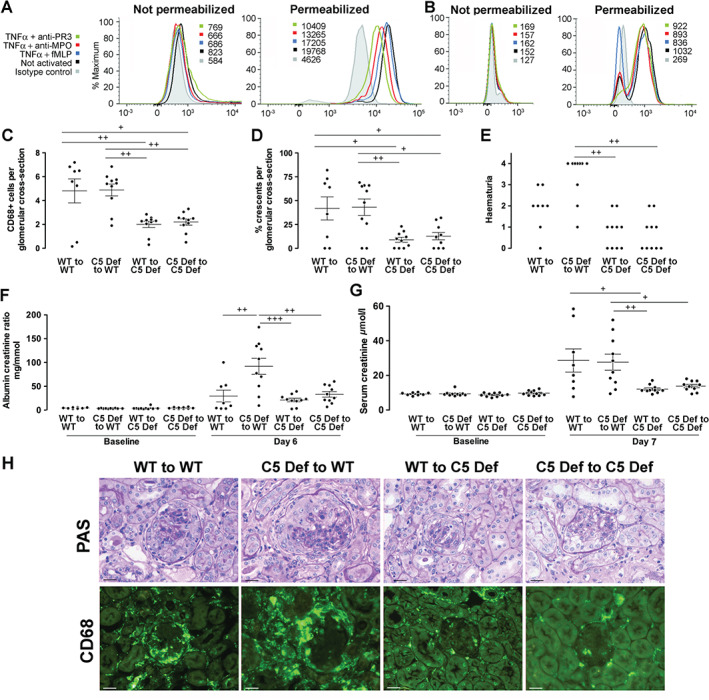
(A) Flow cytometry of isolated human neutrophils showing C5 expression, without and with cell permeabilization, in unstimulated conditions, and after priming with TNFα followed by stimulation with monoclonal anti‐MPO, anti‐PR3 or fMLP. Numbers shown are median fluorescence intensity. (B) Data under the same conditions are shown for C5a staining. Similar results (to panels A and B) were obtained in an experiment from a different neutrophil donor. (C, D) Histological readouts of glomerular CD68+ macrophages and glomerular crescents in anti‐MPO vasculitis in four groups of bone marrow chimaeric mice constructed with wild‐type or C5‐deficient donors and recipients. (E–G) Functional readouts of haematuria, albuminuria, and serum creatinine in the same experiment. For albuminuria and serum creatinine, baseline data from the same animals are shown. Each symbol represents a separate mouse. WT = wild type, C5 Def = C5 deficient. ^+^ *p* < 0.05; ^++^ *p* < 0.01; ^+++^ *p* < 0.001. (H) Representative renal histology showing periodic acid Schiff (PAS)‐stained sections and immunofluorescence staining for CD68+ macrophages from mice in this experiment. Scale bars = 20 µm. Error bars are mean ± SEM.

In view of the emerging data on the importance of intracellular complement components, we explored whether neutrophils contained intracellular C5 and/or C5a, and if so, whether this may play a role in the development of pathology in ANCA vasculitis. We found that small amounts of C5 and C5a were present on the surface of non‐activated neutrophils with a small decrease in C5 expression after priming with TNFα, followed by stimulation with fMLP, anti‐MPO or anti‐PR3 ANCA (Figure 6). Intracellular C5 and C5a were present in greater amounts and decreased after stimulation with fMLP, anti‐PR3 or anti‐MPO. We therefore measured C5a in the supernatant of neutrophils in the absence or presence of the same activating stimuli, in serum‐free conditions. Since released C5a was likely to bind to the C5a receptor, and hence may not be detected in the supernatant, we performed experiments in the presence of PMX53, an antagonist for the C5a receptor CD88. We found somewhat variable results, with C5a undetectable in some donors and levels in the range of 400–600 pg/ml in the presence of PMX53 in others. Monocytes also express C5 and C5a, and this is shown in the supplementary material, Figure S5.

### Circulating and not bone‐marrow derived C5 mediates disease in anti‐MPO vasculitis

We next examined if this intracellular C5a was important in pathogenesis and constructed four groups of bone marrow chimaeric mice from wild‐type or C5‐deficient donors and recipients, and induced anti‐MPO vasculitis. As shown in Figure 6C, D, histological measures of disease showed that crescentic glomerulonephritis was more severe in both groups of C5‐sufficient recipients, compared with both groups of C5‐deficient recipients, as indicated by more crescents and CD68+ macrophages within glomeruli. Functional readouts supported the histology data, as shown in Figure 6E–G. Figure 6H shows representative histology and CD68+ macrophage staining. Circulating neutrophil counts were the same in all groups after GCSF treatment (supplementary material, Table S1). These results show that the key mediator of inflammation in anti‐MPO vasculitis is C5 derived from sources outside of the bone marrow.

## Discussion

The murine anti‐MPO model is an established preclinical model of ANCA vasculitis. This is demonstrated by the fact that the C5a receptor antagonist CCX168 has been tested in this model 12. A phase II trial (NCT01363388) has been completed with CCX168 in patients and a second trial is underway (NCT02222155). Haematuria and proteinuria are variable in this model, as shown by the observation that wild‐type mice had different amounts of haematuria and proteinuria in the experiments shown in Figures 1, 2, 3 and 6. There was also less proteinuria in C5 deficient to wild type than in wild type to wild‐type chimaeras (Figure 6F), despite similar histological findings and serum creatinine. In our experience, the degree of haematuria and proteinuria are not robust markers of disease severity in individual animals and this mirrors the situation in patients with ANCA vasculitis. Histological changes and serum creatinine are therefore the primary readouts, and a lack of correlation with haematuria and proteinuria, if present, would not be a concern. Whilst others have used LPS to obtain robust disease in this model, we also administer GCSF. It is important to consider this when considering our results in relation to those obtained without the addition of GCSF.

We have made a number of observations in this model that are of clinical relevance, given this current interest in targeting complement in patients with ANCA vasculitis. First, the protective role of C3 deficiency has not previously been reported in this model. Here, we have shown for the first time that C3‐deficient mice are protected. It is important to note that we have confirmed that complement is important in this model in our hands, given the lack of protection that we saw in mice deficient in properdin or MASP‐2.

The initiator of AP activation in anti‐MPO vasculitis is not known, and we have excluded both properdin and the lectin pathway as candidates. Both were credible candidates. Activated neutrophils release properdin, which has been suggested as the cause of AP activation by neutrophils 31. In addition to AP activation through properdin binding to apoptotic cells 13, 14, cooperation between properdin and neutrophil‐derived myeloperoxidase may lead to AP activation 32. The lectin pathway was another candidate that had to be excluded because it is a key pathway in other models of kidney inflammation that do not require C4 33. Our findings are important because they pave the way for further studies to focus on other mechanisms of complement activation that may be important. Previously, activation of human neutrophils has been shown to initiate AP activation, although the mechanism of activation was not defined 31. It is therefore possible that neutrophils themselves could be central to AP activation, perhaps via released proteases or induced deficiencies in complement regulatory proteins. Several such factors may combine to lead to AP activation on the surface of neutrophils or perhaps endothelial cells, and further work will be needed to define them.

Disease in MASP‐2‐deficient mice was increased compared with that in wild‐type mice. In view of the previously reported role for the AP, we wondered if the increased disease required complement activation. MASP‐1 has been shown to activate factor D and MASP‐3 can activate factor D and factor B 34, 35. Therefore, the absence of MASP‐2 could result in an increase in MASP‐1 and MASP‐3 circulating in association with MBL and this could potentially enhance AP activation. However, although our results in mice deficient in both MASP‐2 and C3 (Figure 2) were not conclusive, the similarity in the levels of circulating and deposited C3 (supplementary material, Figure S3) in MASP‐2‐deficient mice compared with wild type suggested that the increased disease in MASP‐2‐deficient mice was not due to increased AP activation. Furthermore, wild‐type and MASP‐2‐deficient mouse serum samples were assessed using ELISAs specific to mouse MASP‐1 or MASP‐3. The ELISAs revealed comparable serum levels for MASP‐1 and for MASP‐3 (*n* = 4 per group, data not shown).

A link between coagulation and crescentic glomerulonephritis is well established. Fibrin deposition, initiated physiologically by tissue factor, promotes disease 36, while activation of fibrinolysis through plasminogen activators activating plasminogen is protective 37, 38. We therefore sought an alternative explanation for the increased crescent formation in MASP‐2‐deficient mice and wondered if cross‐talk with the coagulation pathway could be involved. We confirmed that MBL (and hence MASP‐2) is deposited in the glomeruli of mice with crescentic glomerulonephritis, due to anti‐MPO IgG as this would be a requirement for an effect on glomerular fibrin formation. This mirrors data from a recent comprehensive analysis of complement deposition in 187 renal biopsy samples from patients with ANCA vasculitis 39. This study showed that MBL can be detected in over 30% of cases. It may in fact be present in small quantities in a higher proportion of cases, with detection limited by the sensitivity of the staining method.

Activation of either the intrinsic or the extrinsic pathways that make up the coagulation cascade leads to the formation of a prothrombinase complex composed of factors Va and Xa, cleaving prothrombin to thrombin. Thrombin then activates fibrinogen, leading to the deposition of cross‐linked fibrin. Although cleavage of prothrombin by MASP‐2 has been shown to occur at the same molecular site as factor Xa, it is far less efficient 30. Our data show evidence of increased prothrombin activation in MASP‐2‐deficient mice compared with wild‐type mice. Therefore, a plausible explanation for the increase in crescentic glomerulonephritis seen in MASP‐2‐deficient mice is their tendency to enhanced fibrin generation. Despite these findings in coagulation assays, we were unable to detect a difference in fibrin deposition in glomeruli. Whilst this may be due to the relative insensitivity of the immunofluorescence quantification technique used, it is equally possible that the differences in coagulation assays did not lead to a difference in fibrin deposition in glomeruli. The molecular basis for the increased prothrombin activation that we observed has not been established in this study. Further work will be needed to establish precisely how the absence of MASP‐2, which cleaves prothrombin relatively weakly, results in potentiation of the cleavage of prothrombin by the prothrombinase complex.

Circulating complement components are derived primarily from the liver, but it has recently become clear that complement proteins are found not only in serum and plasma but also within leukocytes. Antigen‐presenting cell‐derived C3a and C5a have been shown to augment T‐cell responses 40, with important effects relevant to transplantation and graft versus host disease 16, 17, 18. More recent data have shown that resting human CD4+ T cells contain intracellular stores of C3 and that the protease cathepsin L cleaves C3 into C3a and C3b, with important roles described for T‐cell function and homeostasis 19, 20, 21. Furthermore, the balance of stimulation of the C5a receptors and CD88 and C5L2 affects Th1 T‐cell activation through inflammasome‐mediated IL‐1β secretion 41 and this may be largely due to intracellular C5a (C Kemper, personal communication). However, despite these developments highlighting the role of complement components within leukocytes, our data show that activation of C5 from other sources is important in anti‐MPO vasculitis. Since the synthesis of C5 from murine or human renal cells has not been described, our results demonstrate a role for circulating C5.

The present study includes several observations of direct relevance to developing therapies in ANCA vasculitis. We have shown that the trigger for AP activation is not the lectin pathway or the AP stabilizer properdin, and previous work has excluded the classical pathway 9. Therefore, future work must look for other factors that may initiate cleavage of C3. An ability to target the specific mechanism of complement activation in patients may give more specificity and potentially less toxicity than therapies aimed at C5 or C5a. However, if C5 is to be targeted in patients, an alternative therapeutic approach to C5a receptor blockade or an antibody to C5 such as Eculizumab would be to prevent C5 secretion from the liver. This would not leave intact the important homeostatic interactions between leukocyte‐derived C5a and C5a receptors. Indeed, such an approach is being developed using *N*‐acetylgalactosamine (GalNAc)‐siRNA conjugates to target the hepatocyte asialoglycoprotein receptor. A phase 1–2 clinical trial in patients with paroxysmal nocturnal haemoglobinuria is underway (NCT02352493). The present study supports such an approach in ANCA vasculitis as we have shown that circulating liver‐derived C5 is the key mediator of disease.

## Author contribution statement

SF, RP, MR, CK, and BH conceived the experiments. SF, RP, MK, and KP performed the experiments. All authors (SF, RP, MK, MR, CK, BH, KP, CS, and WS) were involved in data interpretation and writing the paper, and had final approval of the submitted and published versions.

**SUPPLEMENTARY MATERIAL ONLINE**

**Supplementary figure legends**

**Figure S1.** An example of a section stained with only the secondary antibody

**Figure S2.** Anti‐MPO levels measured in serum taken at the end of the experiment where wild‐type and MASP‐2‐deficient mice were compared (shown in Figure 1)

**Figure S3.** (A) Quantitative assessment of glomerular C3 deposition in wild‐type and MASP‐2‐deficient mice with anti‐MPO vasculitis. (B) Circulating C3 levels in untreated wild‐type and MASP‐2‐deficient mice

**Figure S4.** Quantitative assessment of glomerular fibrin and MBL deposition in wild‐type and MASP‐2‐deficient mice with anti‐MPO vasculitis

**Figure S5.** Intracellular staining for C5 and C5a in human blood peripheral blood monocytes

**Table S1.** Neutrophil counts in peripheral blood taken the day before injection of anti‐MPO IgG in each of the experiments shown in this article

## Supporting information


Supplementary figure legends

**Figure S1** An example of a section stained with only the secondary antibody. The same secondary antibody was used for MBL, C3 and CD68 staining (DyLight 488 mouse anti‐rat IgG). In all cases we repeatedly saw negative staining with the secondary only. The glomeruli are easy to see as they are darker than the tubules due to the autofluorescence of the tubules on the PLP fixed sections.

**Figure S2** Anti‐MPO levels measured in serum taken at the end of the experiment where wild‐type and MASP‐2‐deficient mice were compared (shown in Figure 1 of the main article). Each symbol represents data from a separate mouse. There were no differences between groups. Error bars are mean ± SEM.

**Figure S3**. (A) Quantitative assessment of glomerular C3 deposition in wild‐type and MASP‐2‐deficient mice with anti‐MPO vasculitis. (B) Circulating C3 levels in untreated wild‐type and MASP‐2‐deficient mice. Each symbol represents data from a separate mouse. There were no differences in either parameter between groups. Error bars are mean ± SEM.

**Figure S4** Quantitative assessment of glomerular fibrin and MBL deposition in wild‐type and MASP‐2‐deficient mice with anti‐MPO vasculitis. Each symbol represents data from a separate mouse. Error bars are mean ± SEM.

**Figure S5** Intracellular staining for C5 and C5a in human blood peripheral blood monocytes. Monocytes were isolated from peripheral blood by positive selection for CD14 using magnetic beads (Miltenyi Biotec, Bisley, UK) and flow cytometry performed as described for neutrophils. Similar results were obtained in several experiments (at least 3). The isotype control is also shown. We also measure C5a in the supernatants of monocytes cultured in serum free medium at 10^6^ cells/ml for 18 h using monocytes from 2 donors, and found levels of 85–90 pg/ml.

**Table S1** Neutrophil counts in peripheral blood taken the day before injection of anti‐MPO IgG in each of the experiments shown in this article.
