# Supplementary material for: Experimentally‐induced anti‐myeloperoxidase vasculitis does not require properdin, MASP‐2 or bone marrow‐derived C5
Source: J Pathol. 2016 Aug 22;240(1):61–71. doi: 10.1002/path.4754 (PMC4996338; doi:10.1002/path.4754)
Supplement: Supplementary file 1 — Supplementary figure legends [file PATH-240-61-s003.doc]

**SUPPORTING INFORMATION**

**Supplementary figure legends**

**Figure S1.** An example of a section stained with only the secondary antibody. The same secondary antibody was used for MBL, C3 and CD68 staining (DyLight 488 mouse anti-rat IgG). In all cases we repeatedly saw negative staining with the secondary only. The glomeruli are easy to see as they are darker than the tubules due to the autofluorescence of the tubules on the PLP fixed sections.

**Figure S2.** Anti-MPO levels measured in serum taken at the end of the experiment where wild-type and MASP-2-deficient mice were compared (shown in Figure 1 of the main article). Each symbol represents data from a separate mouse. There were no differences between groups. Error bars are mean ± SEM.

**Figure S3.** (A) Quantitative assessment of glomerular C3 deposition in wild-type and MASP-2-deficient mice with anti-MPO vasculitis. (B) Circulating C3 levels in untreated wild-type and MASP-2-deficient mice. Each symbol represents data from a separate mouse. There were no differences in either parameter between groups. Error bars are mean ± SEM.

**Figure S4.** Quantitative assessment of glomerular fibrin and MBL deposition in wild-type and MASP-2-deficient mice with anti-MPO vasculitis. Each symbol represents data from a separate mouse. Error bars are mean ± SEM.

**Figure S5.** Intracellular staining for C5 and C5a in human blood peripheral blood monocytes. Monocytes were isolated from peripheral blood by positive selection for CD14 using magnetic beads (Miltenyi Biotec, Bisley, UK) and flow cytometry performed as described for neutrophils. Similar results were obtained in several experiments (at least 3). The isotype control is also shown. We also measure C5a in the supernatants of monocytes cultured in serum free medium at 10^6^ cells/ml for 18 h using monocytes from 2 donors, and found levels of 85-90 pg/ml.
