# Supplementary material for: Experimentally‐induced anti‐myeloperoxidase vasculitis does not require properdin, MASP‐2 or bone marrow‐derived C5
Source: J Pathol. 2016 Aug 22;240(1):61–71. doi: 10.1002/path.4754 (PMC4996338; doi:10.1002/path.4754)
Supplement: Supplementary file 7 — Table S1 Neutrophil counts in peripheral blood taken the day before injection of anti‐MPO IgG in each of the experiments shown in this article. [file PATH-240-61-s004.docx]

**SUPPORTING INFORMATION**

**Table S1.** Neutrophil counts in peripheral blood taken the day before injection of anti-MPO IgG in each of the experiments shown in this article. Neutrophil counts at ×10^7^/ml. There were no significant differences between groups in any of the experiments

| **Wild type** | **MASP-2 def** |  |  |
| --- | --- | --- | --- |
| 1.28 ± 0.09 | 1.15 ± 0.06 |  |  |
| **Wild type** | **MASP-2/C3 def** | **C3 def** |  |
| 0.68 ± 0.04 | 0.65 ± 0.1 | 0.65 ± 0.08 |  |
| **Wild type** | **Properdin def** |  |  |
| 1.41 ± 0.09 | 1.31 ± 0.05 |  |  |
| **Wild type to Wild type** | **C5 def to**  **Wild type** | **Wild type to C5 def** | **C5 def to**  **C5 def** |
| 1.62 ± 0.11 | 1.75 ± 0.07 | 1.86 ± 0.06 | 1.96 ± 0.1 |
